# Codeveloping a Virtual Patient Simulation to Foster Nurses’ Relational Skills Consistent With Motivational Interviewing: A Situation of Antiretroviral Therapy Nonadherence

**DOI:** 10.2196/18225

**Published:** 2020-07-15

**Authors:** Geneviève Rouleau, Jérôme Pelletier, José Côté, Marie-Pierre Gagnon, Valérie Martel-Laferrière, Rock Lévesque, SimforHealth, Guillaume Fontaine

**Affiliations:** 1 Research Chair in Innovative Nursing Practices Montréal, QC Canada; 2 Faculty of Nursing Université Laval Quebec, QC Canada; 3 Université du Québec à Rimouski Rimouski, QC Canada; 4 University of Montreal Hospital Research Centre Montreal, QC Canada; 5 Faculty of Nursing Université de Montréal Montreal, QC Canada; 6 University Hospital Centre of Quebec Laval University Research Centre Quebec, QC Canada; 7 Institute of Health and Social Services in Primary Care Research Centre on Healthcare and Services in Primary Care Quebec, QC Canada; 8 University of Montreal Hospital Centre Montreal, QC Canada; 9 Please see acknowledgements for a list of collaborators; 10 Research Centre Montreal Heart Institute Montreal, QC Canada

**Keywords:** motivational interviewing, HIV, nurses, education, continuing, virtual patient, simulation, nurse-patient relations, communication

## Abstract

**Background:**

Although helping people living with HIV manage their antiretroviral therapy is a core competency of HIV nursing care, no educational intervention has sought to strengthen this competency. Thus, we codeveloped a simulation of a virtual patient (VP) having difficulty adhering to treatment to foster the relational skills that nurses require in such situations.

**Objective:**

This viewpoint paper aims to describe the codevelopment process and the content of VP simulation, as well as the challenges encountered and the strategies used to overcome them.

**Methods:**

We use a collaborative and iterative approach to develop the simulation based on qualitative evidence, theoretical approaches (strengths-based nursing, motivational interviewing [MI], and adult learning theories), and expert recommendations. We carried out 2 main phases: (1) planning the simulation development and (2) designing the simulation content, sequence, and format. We created the script as if we were writing a choose-your-own-adventure book. All relational skills (behavior change counseling techniques derived from MI) were integrated into a nurse-patient dialogue. The logic of the simulation is as follows: if the nurse uses techniques consistent with MI (eg, open-ended questions, summarizing), a dialogue is opened up with the VP. If the nurse uses relational skills inconsistent with MI (eg, providing advice without asking for permission), the VP will react accordingly (eg, defensively). Learners have opportunities to assess and reflect on their interventions with the help of quizzes and feedback loops.

**Results:**

Two main challenges are discussed. The most salient challenge was related to the second phase of the VP simulation development. The first was to start the project with divergent conceptions of how to approach the VP simulation—the simulation company’s perspective of a procedural-type approach versus the clinical team’s vision of a narrative approach. As a broad strategy, we came to a mutual understanding to develop a narrative-type VP simulation. It meshed with our conception of a nurse-patient relationship, the values of strengths-based nursing (a collaborative nurse-patient relationship), and the MI’s counseling style. The second challenge was the complexity in designing realistic relational skills in preprogrammed and simulated nurse-patient dialogue while preserving an immersive learning experience. As a broad strategy, we created a collaborative and work-in-progress writing template as a shared working tool.

**Conclusions:**

Our experience may be helpful to anyone looking for practical cues and guidance in developing narrative VP simulations. As relational skills are used by all nurses—from novices to experts—and other health care practitioners, focusing on this clinical behavior is a good way to ensure the simulation’s adaptability, sustainability, and efficiency.

## Introduction

### The Role of Simulation in Continuing Professional Development

Professional expectations and social accountability require nurses to pursue continuing professional development (CPD) to reinforce and maintain their competencies, so they can provide evidence-based care and ensure patient safety [1,2]. CPD is typically offered through interactive and/or didactic educational meetings such as conferences, workshops, seminars, lectures, and courses [3]. However, many of these approaches require time, money, and human resources, which are often limited in health care environments [4,5]. There is a need to develop accessible and innovative ways to strengthen nurses’ learning and expertise while considering resource availability and workplace realities. The use of virtual patient (VP) simulation, a digital learning modality, is one way to address these challenges and is increasingly used in contemporary nursing education [6].

### Virtual Patient Simulation and Education for Health Professionals

VP simulation is an interactive computer simulation that depicts real-world scenarios with the goals of training, education, and assessment [7] for health care professionals. A VP enables learning in a nonjudgmental, ethical, and safe environment [4,8-10] as learners acquire knowledge and develop skills by learning from their mistakes, without harming patients [11]. The use of VP simulation in health professional education is growing exponentially, considering the number of reviews published in the field [6,12-15]. Although the populations included in these reviews are mainly undergraduate and postgraduate students and health care providers in medicine, nursing, and other disciplines, we found only 1 integrative review focusing exclusively on web-based simulation in nursing programs [6]. Thus, it is relevant to explore the use of VP simulation as a means of supporting CPD for nurses.

On the basis of the needs identified by nurses providing HIV care in a qualitative study [16], we developed a VP simulation aimed at improving the nurses’ relational skills consistent with motivational interviewing (MI) [17] in situations of antiretroviral therapy nonadherence. MI is a person-centered, collaborative communication style that seeks to elicit people’s motivation and commitment to change. A systematic review conducted by Shingleton and Pafai [18], which included 41 studies, aimed to characterize the use of technology-delivered MI interventions and their efficacy at changing various health-related behaviors among several populations. One of the authors’ recommendations was to provide details, for instance, in methodological papers, on how the relational (eg, empathy) and technical (eg, confidence rulers) components of MI are translated into a technology-delivered intervention.

### Insufficient Guidance for Developing a Specific Type of Educational Intervention

Several approaches exist to develop interventions aimed at improving health [19] and supporting behavioral change by health care professionals [20]. Relative to a taxonomy of eight approaches to intervention development [19], we locate ours as a specific type of intervention and behavior [21] because of the following elements: its modality (VP simulation), the action to be performed (adoption of relational skills consistent with MI), the actors performing the action (nurses and, more broadly, all health care professionals), the target behavior (medication adherence), and the recipients of this action (patients who are living with HIV or not). Developing such a specific intervention was challenging, given that we faced a scarcity of operational and practical guidelines for doing so, particularly with regard to creating and translating the relational skills into preprogrammed schemes of communication between the virtual nurse and VP. Although we strongly believe in the value of development frameworks [19,20] to broadly inform intervention development, these frameworks are not intended to offer the extensive and practical description needed to guide the creative process of translating relational skills into an intervention-specific VP simulation. Furthermore, while the evaluation of interventions is well known to be a core research activity, the interventions themselves and their methods remain poorly described in the literature. Cheng et al [10,22] supported the importance of providing a detailed description of the development process of simulation.

### Relevance of This Viewpoint Paper

This viewpoint paper attempts to fill the gaps cited earlier. In addition, this study may be helpful to VP simulation developers, educators responsible for CPD, and health care professionals wanting to undertake such an endeavor. At the same time, as our paper ensures transparency in reporting the development methods, the components of the VP simulation (including its content), and the lessons learned, it provides insights into an approach to codeveloping a theory-informed VP simulation aimed at improving nurses’ relational skills with patients. This viewpoint paper provides practical cues on how to deliver and translate MI through technology into preprogrammed nurse-patient dialogues.

### Scope, Aim, and Objectives

This viewpoint paper is an opportunity to make explicit the tacit knowledge that comes from our personal experience in codeveloping a VP simulation, supported by theoretical approaches and the literature. The specific objectives are to describe (1) the entire codevelopment process of a VP simulation, including its guiding principles, its content, and features and (2) the challenges encountered and the strategies used to overcome them.

## Methods

### Codeveloping a Virtual Patient Simulation and Its Content

In this section, we describe the methods and processes involved in 2 main phases: (1) planning the VP simulation development and (2) designing the content, sequence, and format of the VP simulation. We also identify the deliverables (or outputs) of each phase and subphase, such as the qualitative evidence of the needs assessment, the composition of an interprofessional team, the clinical content, and the graphical presentation of the VP simulation. We used a collaborative and creative approach to codeveloping the VP simulation. The term *codevelop* is preferred because 2 nurses worked together to produce the clinical content from scratch, along with the project manager of the VP simulation team, and with the support of a larger interprofessional team. Some models [23,24] and standards of best practice in simulation [25] inspired and broadly informed the general codevelopment processes. Although being very useful, especially in the planning phase, these models did not provide operational guidance for the design phase, particularly with regard to creating the full conversational script of the nurse-patient consultation rooted in relational skills. Therefore, describing this process of codevelopment was a retrospective exercise: it was not preplanned but was rather emergent.

The processes are presented in a linear manner to facilitate understanding. However, in reality, they were conducted concurrently, and some in a preordered sequence (the planning phase was done before the design phase). We used field notes, meeting summaries, and debriefing sessions with team members to keep track of important decisions throughout the project. In this paper, sentences or groups of words in *italics* are used to emphasize important guiding principles or key elements surrounding the codevelopment of the VP simulation.

### Phase 1: Planning the Virtual Patient Simulation Development

There are 4 subphases related to the planning phase: (1.1) assessing training needs; (1.2) selecting theoretical approaches to inform the VP simulation development; (1.3) negotiating a detailed partnership contract between the research institution, the researcher, and the VP simulation company; and (1.4) assembling an interprofessional team.

#### 1.1. Assessing Training Needs by Understanding HIV Nursing Practice and Its Challenges

To gain insight into HIV nursing practice and the challenges nurses face when providing antiretroviral therapy–related care, a qualitative study [16] was conducted, identifying 3 challenges. The first was performing nursing roles at the interface of social and biomedical boundaries: nurses sometimes felt unequipped to perform their social role. The second challenge was the lack of alignment in the expectations of nurses and patients regarding antiretroviral therapy adherence. The third focused on dealing with the sociopolitical determinants affecting access to health care resources and services.

From these results, *one challenge was chosen and prioritized because it targeted a clinical behavior amenable to change within the nurse-patient relationship and offered* *room for practice improvement and change*: the misalignment between the expectations of nurses and patients regarding medication adherence. Some nurses had an optimistic attitude toward antiretroviral therapy; they encouraged patients to take their medication while expecting an outcome of adherence that was not achieved. Nurses felt powerless in such situations of nonadherence, as though they had failed in their role. One of the strategies used by nurses to overcome this challenge was to use the MI [17] approach. This challenge was then transformed into a learning opportunity and thus appeared feasible for translation into a simulated clinical situation.

#### 1.2. Selecting Theoretical Approaches to Inform Virtual Patient Simulation Development

We selected 3 theoretical approaches to lay the groundwork for the VP simulation, each serving a different purpose. First, strengths-based nursing (SBN) was chosen as a philosophy and value-driven approach [26] to clarify the goals and mission of nursing overall. *It offers a lens through which to view* *the roles of nurses and patients, the focus of nursing care, and the nature of the nurse-patient relationship. The assumptions and values underlying SBN guided the conceptualization of the core of the simulation to be a collaborative partnership within the nurse-patient relationship.* Second, while SBN allows for a broad conception of the role of nurses, *MI is the approach used to design the virtual nurse’s key actions in the simulation. MI was used to inform the creation of the VP simulation content (ie, the nurse-patient dialogue, quizzes, and feedback)*. The use of MI aimed to operationalize content creation. Third, *considering the learner-centered vision of the VP simulation, the principles of adult learning theories were identified to support the learning processes and activities*. SBN and adult learning theories were used for the basic structure of the VP simulation.

#### Strengths-Based Nursing Approach

SBN is grounded in the principles of person and family-centered care, empowerment, relational care, and innate health and healing. *Collaborative partnership,* one value underlying SBN, meshes with our conception of the nurse-patient relationship. The term *collaborative partnership* refers to power, to the way it is distributed, and how it is shared with the person/family to give them a voice in achieving their goals [26]. In this approach, goals are set by the nurse and patient together, each bringing their own experience, knowledge, and competencies to the relationship [27]. Under a facilitator role, the nurse provides guidance to help patients find their own solutions. The recognition of the patient’s strength is a key element in this collaborative relationship.

In Textbox 1, we present the criteria underlying the collaborative partnership adapted from SBN [28], showing how this form of the nurse-patient relationship can guide nursing practice based on different assumptions, principles, and values. The purpose here is to raise awareness and reflect on how nursing activities and actions can potentially be influenced or shaped by broad conceptions of the nurse-patient relationship, whether these conceptions are known or unknown, conscious or unconscious, implicit or explicit.

**Collaborative partnership within the nurse-patient relationship; strengths-based care.**

**Focus**

The person’s capacity to be well, have a high quality of life, and experience it in a meaningful manner

**Role of the nurse**

The nurse is a facilitator. Encourages people to share their perspectives and expertise, participate in shared decision-making processes, develop their autonomy, and use their strengths and resources

**Role of the person (includes patient/client and the family)**

Acts as an active partner who plays an important role in setting goals and in looking for solutions that best match the person

**Nature of the relationship**

It is reciprocal and mutual, more symmetrical, with continuous negotiation of goals, roles, and responsibilities. Both partners give and receive and gain and grow

**Goal setting**

Goals are jointly set

**Evaluation**

Nurse and person share in the joint assessment of progress in reaching mutually determined goals

**Expected outcome**

The problem may or may not be solved, but the person’s skills for managing current or future problems are reinforced. Joint responsibility is accepted for the outcomes

#### Motivational Interviewing

The SBN approach and MI share common roots and a core value: collaborative partnership. The person-centered communication style characterizing MI [17] can be established only in a relationship where these 4 values are present: partnership, acceptance, evocation, and compassion. In MI, collaborative partnership is about honoring and respecting the person’s autonomy and seeking to understand the person’s internal frame of reference.

In addition to these values, the VP simulation is based on a number of elements related to MI. First, the VP simulation is divided into 4 processes: (1) engaging, (2) focusing, (3) evoking, and (4) planning (see definitions given in Textbox 2).

**Definitions of the 4 motivational interviewing processes that are the building blocks of the virtual patient simulation.**

1. *Engaging:* “Engaging is the process of establishing a mutually trusting and respectful helping relationship” [17]. Engaging in the relationship can be very brief at times, as is the case in the consultation between Mr Wilson and the nurse who have known each other for a long time. At other times, engaging can be very long. However, in all cases, it goes beyond stock greetings or courtesy. Engaging must be manifest throughout the therapeutic relationship. It is about the development of a *working alliance* [17]
2. *Focusing:* Focusing serves to pinpoint the goal to be achieved to set and maintain a direction. To this end, nurses clarify the following with patients:
  - Their values and goals
  - The nature of the desired change
  - The importance patients attribute to the change
3. *Evoking:* “Evoking involves eliciting the client’s own motivation for change” [17]. At this stage, nurses guide patients in exploring their motivation, while simultaneously evoking hope and confidence. To this end, nurses:
  - Explore with patients their perceptions (*ambivalence*) about change
  - Mobilize relational skills to elicit *change talk* (a discourse in favor of changing the patient’s health-related behavior such as medication adherence)
  - Mobilize relational skills to respond to *sustain talk* (a patient’s discourse in favor of the status quo)
4. *Planning*: A clear plan drawn up by the patient and guided by the nurse is conducive to engagement toward change and success in effecting change. To this end, nurses:
  - Recognize signs or cues indicating that patients are ready to take action (ie, increased *change talk or* decreased *sustain talk*)
  - Avoid slipping (back) into a *directing counseling style* by drawing up the plan for the patients
  - Make a transition from *evoking* to *planning* (eg, “Where does this get you?” “What do you intend to do about it?” and “Let’s imagine for a moment that you decide to change: How might you go about it?”)
  - Mobilize their relational skills and their practical knowledge to clarify the plan
  - Guide patients in anticipating obstacles and solutions to these

Second, the VP simulation is based on relational skills that are essential to the proficient practice of MI (Multimedia Appendix 1) by creating optimal conditions for relational engagement with patients: asking open-ended questions, using reflective listening, summarizing, affirming the patients’ strengths, providing information and advice, evoking a hypothetical change, eliciting and scaling change talk, setting patient-determined goals, and arriving at a plan. These relational skills are called *behavior change counseling techniques* because they are active ingredients that allow providers to initiate or maintain health behavior changes [29-31]. Third, traps or roadblocks (eg, expert and blame traps, a directing counseling style) are also part of the VP simulation (Multimedia Appendix 2). These are interventions, advice, or relational skills inconsistent with MI that are delivered by nurses with the best of intentions to help their patients but are likely to cause relational disengagement and to shut down dialogue with them.

Several reasons justify the use of MI to inform the VP simulation. First, some nurses from the previous work used it in their current practice and found it effective, whereas others clearly expressed the need for training in it [16]. Second, MI training is promising for improving the relational skills of health care professionals working in HIV care [32] and has proven effective in enhancing medication adherence [33-35]. Third, MI is commonly used as a behavioral change counseling approach in nursing, as highlighted in a systematic review conducted by Fontaine et al [30]. This means that some nurses may be familiar with it and associate this approach with prior knowledge and experience.

#### Adult Learning Theories

Simulation is rooted in certain principles of adult learning theories [36] that describe how adults learn and gain an understanding of clinical expertise. In our case, [37,38] is created by the interaction of nurses with the VP simulation environment. The learners, or nurses (these terms will be used interchangeably), will be actively involved in the simulated experience by practicing and testing relational skills with the VP. They will have opportunities to assess their interventions and reflect on them with the help of quizzes and feedback loops (these will be explained later on). We sought to create a *transformative learning experience* [39]. *Transformative learning* capitalizes on the learners’ prior experiences and their interpretation of a situation to build a new way of thinking and then act differently by using critical reflection [36,40]. Feedback is a powerful and effective mechanism to support adult learning and was integrated throughout the VP simulation [41].

*In sum, SBN and adult learning theories contributed structuring elements with regard to the goal of the nurse-patient relationship and learning processes. MI informed the integration of concrete communication techniques in the fine content of the VP simulation.*

#### 1.3. Negotiating a Detailed Partnership Contract Between the Research Institution, the Researcher, and the Virtual Patient Simulation Company

The coming together of the research team and a representative of the VP simulation company (SimforHealth) was an important aspect of this project. The research team was composed of experts in clinical content, and the company had expertise in digital training for health care providers [42]. A person with extensive experience at the research institution was in charge of negotiating the terms of the contract between the researcher (JC) and the company. These terms were financial considerations, accountability and commitments, mandate duration and timeline, confidentiality duties, intellectual property, and platform use licenses.

#### 1.4. Assembling an Interprofessional Team

An interprofessional team was formed, comprising experts from a VP simulation team and clinical, research, and community-based settings. The VP simulation team was composed of a project manager, a pedagogical engineer, two-dimensional (2D) design professional, 3D graphic designers, and a software engineer. The clinical, research, and community team members included nurses (clinical nurse specialists, a head nurse, researchers, and student researchers, including one with experience using MI with people living with HIV), an infectious disease specialist, the director of an HIV community-based organization, a woman living with HIV, and researchers with experience in developing and evaluating web-based interventions to improve antiretroviral therapy adherence and in health technology assessment.

Furthermore, 2 members of the team (GR and JP) cocreated the clinical content of the VP simulation and worked in close collaboration with the project manager of the VP simulation team. These 3 people made up the working committee. The other team members played a consultative role, providing input on the clinical content and graphical components (eg, the VP, the nurse’s office).

### Phase 2: Designing the Content, Sequence, and Format of the Virtual Patient Simulation

This second phase includes the following subphases: (2.1) setting the learning objectives and cocreating the clinical content; (2.2) recording the nurse and patient voice-overs; (2.3) designing and validating the 2D learning environment; and (2.4) integrating 3 modes of fidelity to ensure learner engagement and immersion in the VP simulation.

To provide an exhaustive description of the content, sequence, and format of the VP simulation and of how we can translate MI through technology, we have provided detailed information on the following in Multimedia Appendices 1 to 5: the relational skills that are or are not consistent with MI, the key elements of the VP simulation, an excerpt of the writing template, and the table of contents of the glossary. In Multimedia Appendix 3, we offer a comprehensive description of all the key elements (eg, simulation designs such as quizzes and feedback points, learner orientation, exposure, participant groups) constituting the VP simulation, which was adapted from Cheng et al [10,22] and Peddle et al [43].

#### 2.1. Setting the Learning Objective and Cocreating Clinical Content

The learning objectives evolved throughout the clinical codevelopment of the VP simulation and are listed in Multimedia Appendix 3. Cocreating the clinical content involved more than just the wording of the script for the nurse-patient dialogue. It was a creative process that took into account the assumptions, nursing philosophy, and values embedded in the simulated situation (ie, SBN)—the active ingredients of MI and the learners’ roles (adult learning theories).

The clinical content as a whole was made up of the following: a prebriefing video featuring the nursing student-researcher and its corresponding text; the simulated clinical situation, also referred to as the “virtual nurse-patient consultation,” which includes the patient’s electronic record (also named patient’s file); a glossary; quizzes and feedback loops; and labels (green and red visual cues).

*Key simulation design elements and features, including prebriefing, repetitive practice, MI techniques, quizzes, feedback, labels, and fidelity, were included to optimize interactivity, foster nurses’ engagement with and immersion in the training, and to promote their active and transformative learning.*

#### Prebriefing

The simulation-based experience with a prebriefing is considered the best practice in simulation [25]. In our case, the goal of the prebriefing, provided via video and text, was to offer basic information about MI so that all learners could begin the simulated nurse-patient consultation with the same standardized information. It also aimed to set boundaries about the scope of the simulation to help nurses manage their expectations. For instance, some nurses might otherwise have thought that the simulation’s goals were to acquire or deepen knowledge of antiretroviral therapy, or that prior experience with people living with HIV was needed (neither of which were the case). The prebriefing was also an opportunity to establish a fiction contract, which refers to a kind of commitment in which nurses are invited to act as though the simulation is real, although acknowledging its limitations [44,45]. Multimedia Appendices 6 and 7 show excerpts of the prebriefing video and a demonstration of the VP simulation.

#### Patient Electronic Record

**Figure 1:**
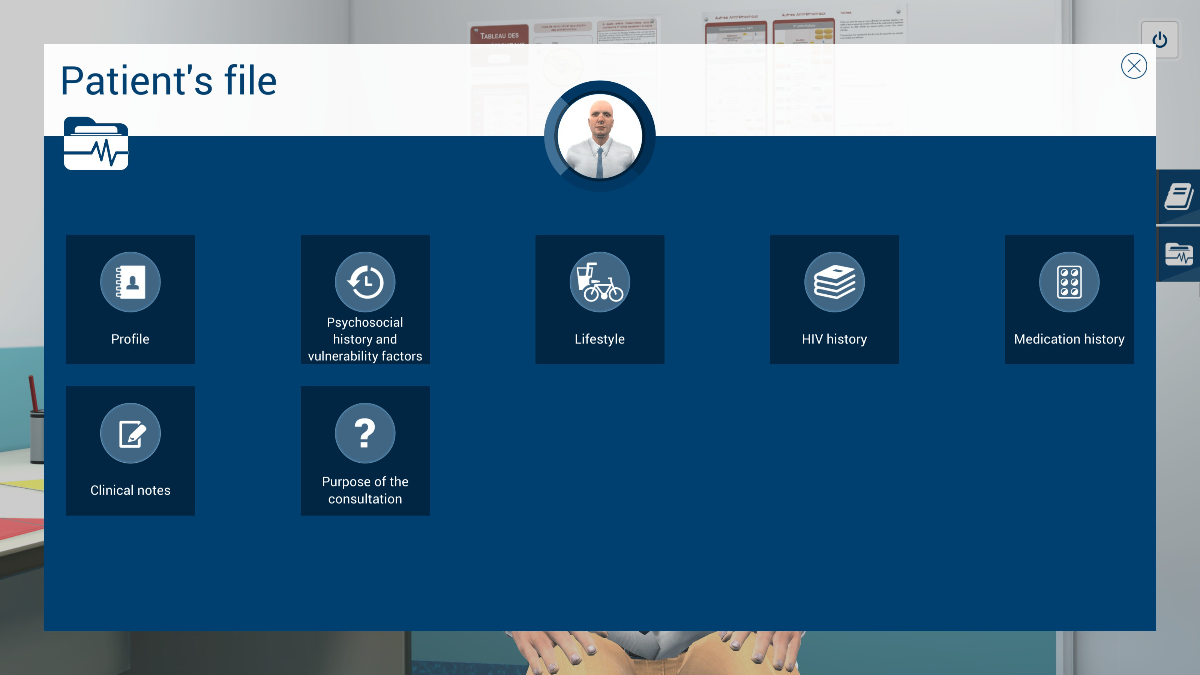
Patient's electronic file rubrics.

The content of the patient’s electronic record was cocreated with the interprofessional team members for 2 reasons: (1) to ensure the credibility and validity of the patient’s medical history and (2) to ensure the fidelity with real patient record rubrics in HIV outpatient clinics. The purpose of the patient’s consultation is presented in the patient’s record and is summarized in Textbox 3. The different rubrics of the patient’s electronic record (eg, clinical notes, HIV, and medication history) are illustrated in Figure 1.

**Purpose of consultation.**

The story is about Mr Wilson, a 50-year-old man living with HIV since 2011. He generally takes his medication regularly. His viral load was undetectable for 6 years (which is a sign of antiretroviral therapy adherence). Mr Wilson changed his treatment 1 year ago. He was very busy at work, and his routine changed, so it was difficult to take the medication as prescribed. Now, his viral load is over 1000 copies/ml, indicating that the antiretroviral therapy is not as effective (the target is to achieve an undetectable viral load, that is, below 40 copies/ml). What is going on?

#### Full Script of Nurse-Patient Consultation

We created the script as if we were writing a *choose-your-own-adventure* book. We started by writing the *green pathway*, that is, the whole storyline in which the script is based on relational skills that are consistent with MI. Afterward, we identified strategic places in the script to incorporate traps or roadblocks—the *red pathways*. These crossroads between the green and red pathways are introduced by quizzes in which learners must select an intervention. When a red pathway is chosen, a short interaction inconsistent with MI shows learners how some types of communicational traps used by nurses generate negative reactions in the patient’s speech. At the end of the interaction, the red pathway stops, written feedback is given to the learners, and they can go back to the crossroads to make a different choice. When the green pathway is chosen, the script (ie, nurse-patient dialogue) becomes automated until the next crossroads. We had to carefully select the strategic places within the script to insert the crossroads. Thus, the arborescence that supports the whole script is made up of the green and red pathways, including quizzes and feedback loops.

*All these relational skills, whether they are consistent with MI (eg, open-ended questions) or not (eg, providing advice without asking for permission), were integrated into a nurse-patient dialogue in which learners had to choose between the interventions that generate openness in the patient’s speech and those that can shut down communication. This scenario provides learners with safe, constructive, positive, and nonjudgmental spaces that allow for transformative learning and self-reflective practice * [36].

The main stages in writing, validating, and producing (ie, filming, voice-over recording) the overall content and testing it on the platform are summarized in Textbox 4. Steps 1 to 4 are more general and account for how we created a script for nurse-patient consultation.

In our various attempts to write the script, we developed multiple versions of a writing template. This was a work in progress throughout. The final template (Multimedia Appendix 4) includes the nurse-patient dialogue (script), quizzes, feedback, labels, guidelines for the VP simulation team regarding the branching, and notes for the actors who would record the voice-overs. This template was a tool that allowed us to write the script in the manner of a *choose-your-own-adventure* book.

A total of 14 quizzes, including multiple-choice and open-ended questions, are part of the VP simulation. Most were formulated to allow learners to select the most appropriate intervention, depending on the script’s progress and the patient’s speech. Each quiz is supported by synchronous written feedback focusing on the rationale for the good or bad answer selected based on their consistency with or without MI (Multimedia Appendices 1 and 2).

*Visual cues*, called *green* and *red labels*, were introduced during the nurse-patient consultation to qualify their speech (eg, open-ended questions, defensive attitude) and in the written feedback. These labels correspond to theoretical MI techniques and provide feedback. The introduction of visual cues into the automated dialogue allows learners to grasp the rationale for the virtual nurse’s communication skills and to observe the patient’s reaction while limiting the number of pathways (green and red) to be scripted.

**Main stages in writing, producing, and validating the overall content and making it accessible as a virtual patient simulation platform.**

1. Writing the green pathways, meaning the overall storyline between the nurse and patient that is consistent with motivational interviewing, by using behavior change counseling techniques (Multimedia Appendix 1), and structured according to its 4 processes: (1) engaging, (2) focusing, (3) evoking, and (4) planning (Textbox 2). *Note:* The script of the nurse-patient dialogue was first written in a linear way to open up onto the patient’s experience. At this stage, no crossroads (red pathways) were part of the script
2. Validating the green pathway with the interprofessional team
3. Identifying strategic places along the green pathway where traps or roadblocks could be integrated and then writing the *red pathways* (Multimedia Appendix 2). This step included the creation of quizzes and feedback
4. Creating visual cues that we called *green* and *red labels* to engage the learner observing the preprogrammed dialogue. These labels were integrated into strategic places in the script (eg, when the nurse uses a directing counseling style)
5. Producing the content of the patient’s electronic record, the glossary, and the prebriefing (video and text)
6. Validating the whole clinical content with members of the interprofessional team
7. Culturally adapting the script (nurse-patient dialogue). Originally, it was written in French from Quebec (Canada). The text was adapted to *international* French
8. Recording of the nurse’s and the patient’s speech by professional actors and filming the prebriefing video
9. Integrating all the content in the MedicActiV platform [42]
10. Performing functionality tests and validating the content within the platform
11. Validating the virtual patient simulation with a small group of nurses
12. Launching the French version
13. Translating the virtual patient simulation to English and revising the content in close collaboration with an anglophone nurse who was an expert in motivational interviewing and a member of the Motivational Interviewing Network of Trainers.

*Note:* Steps 9 to 13 were repeated for the English version.

#### Glossary

A web-based glossary is available within the VP simulation as supplementary educational material to complement the content of the nurse-patient consultation. It covers theoretical concepts, definitions, and applications. The main topics covered in the glossary are presented in Multimedia Appendix 5.

#### 2.2. Recording the Nurse and Patient Voice-Overs With French- and English-Speaking Actors

The project manager of the VP simulation team was responsible for preselecting French- and English-speaking actors and assisting them during the recording of the nurse and patient voice-overs.

*It is important to add stage directions in the writing template, alongside the speech of the nurse and patient, so that the actors can respect the tone of voice and the ambience/vibe in the virtual nurse-patient relationship, consistent with MI.*

The nurse’s mode of communication had to be respectful, calm, warm, and welcoming, without becoming caricatured, especially in the red pathways. That way, the learners could not guess or deduce the right answer merely from the character’s tone of voice or an intervention that is obviously inadequate. As for the patient, if he was feeling worried, the actor had to convey this in his performance.

#### 2.3. Designing and Validating the 2D Learning Environment

**Figure 2:**
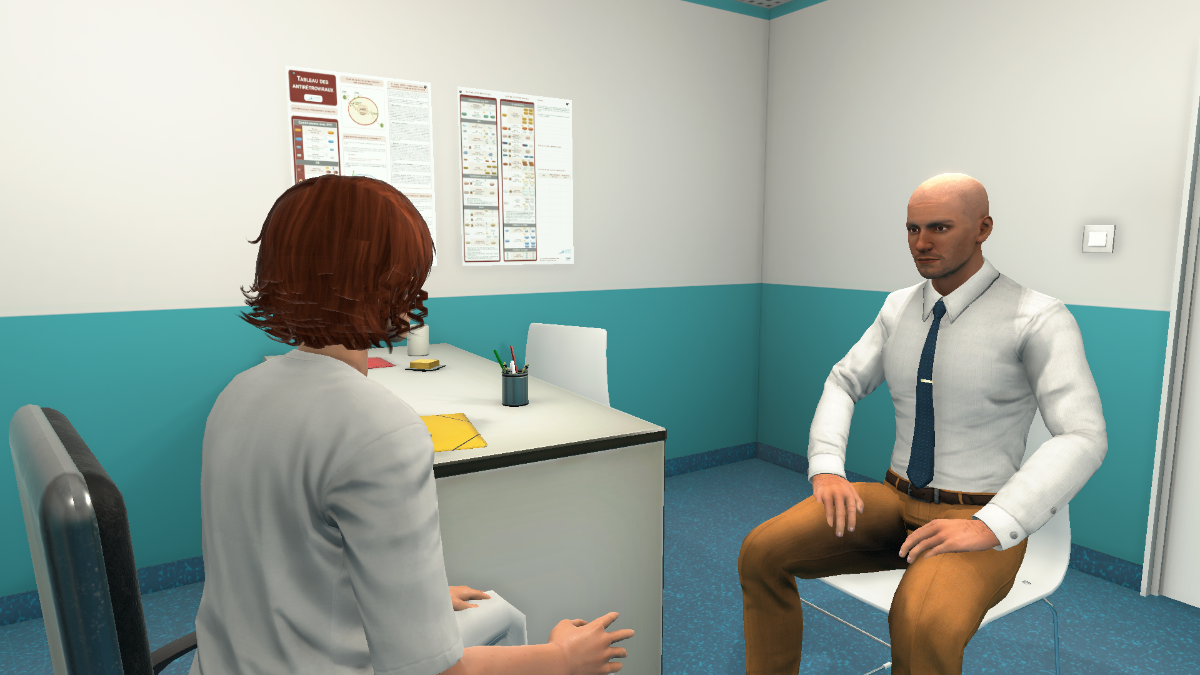
Mock-up of the virtual office including nurse and patient.

**Figure 3:**
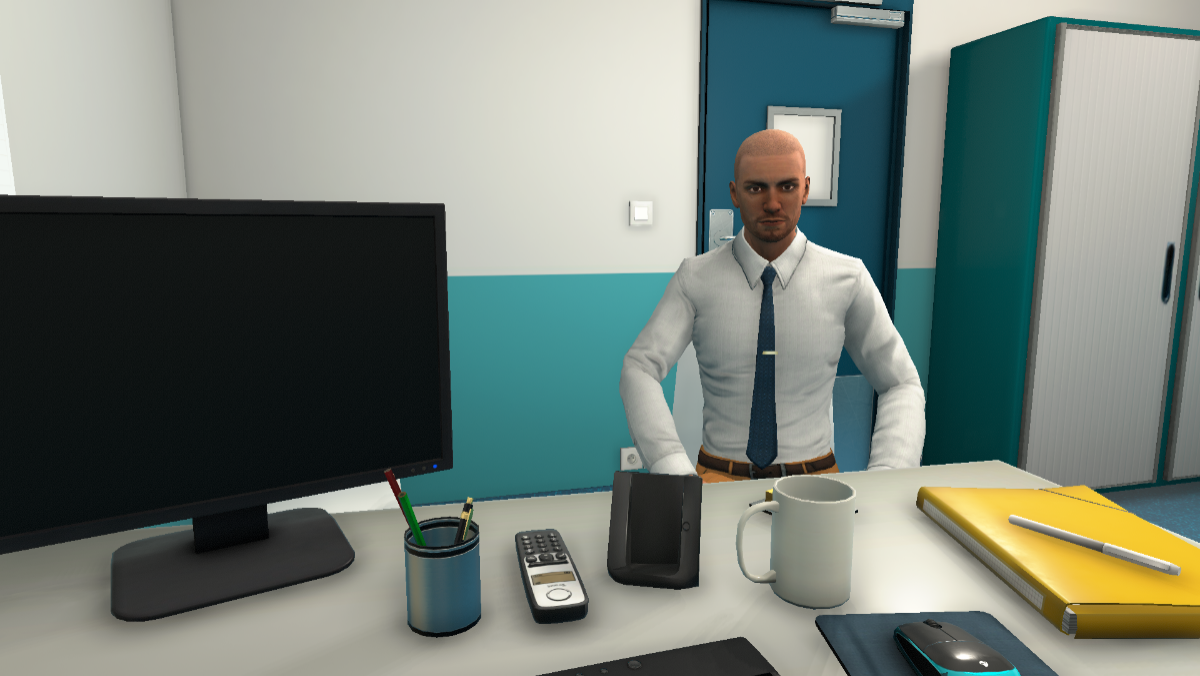
Mock-up of the virtual patient.

At the same time, as the clinical content was being written, the VP simulation team was working on designing the graphical elements of the 2D learning environment. Figure 2 shows a mock-up of the virtual office, including both nurse and patient, and Figure 3 shows the patient only.

**Figure 4:**
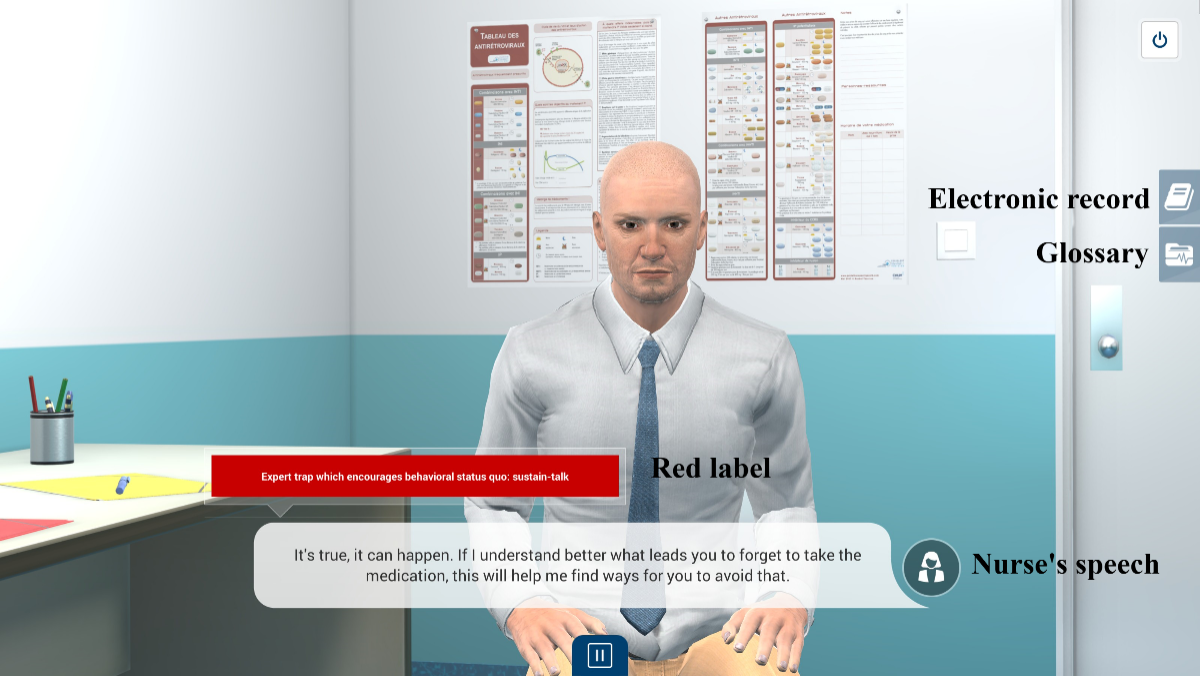
Final design of the virtual patient simulation.

These mock-ups allowed us to obtain the team members’ opinions of the graphical designs before selecting the final 2D learning environment. It was important to display a chart of antiretroviral therapy on the wall to represent a real-life artifact. Decisions about the virtual nurse and patient had to then be made. For example, would we see the nurse on-screen (third-person view) or not (first-person)? What would the HIV-positive man look like? How would the desk be positioned in the virtual office (ie, between the nurse and patient or on the side)? Ultimately, the first-person view was preferred, where the nurse is not visible on-screen, but we hear her voice and see her speech. In this way, only the VP is visible. The desk was placed on the side to avoid creating distance between the nurse and patient. Figure 4 shows the final design of the virtual nurse’s office and the patient (Mr Wilson).

Some features were created to fit with this project, such as the green and red labels, and the interfaces to support synchronous feedback. Furthermore, SimforHealth developed a specific content and dialogue management module (questions/answers) to meet the clinical team’s needs. This module made it possible to speed up the content creation (full script of the nurse-patient consultation) and facilitated content integration into the VP simulation.

**Figure 5:**
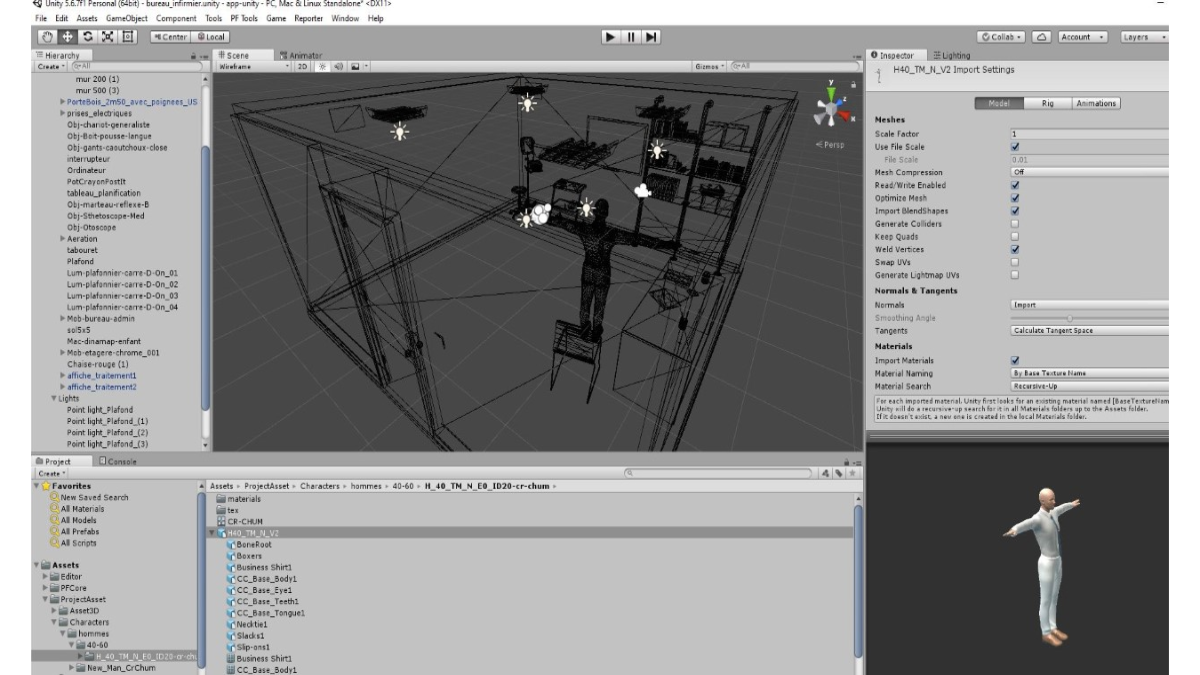
3D digital content creation solution.

**Figure 6:**
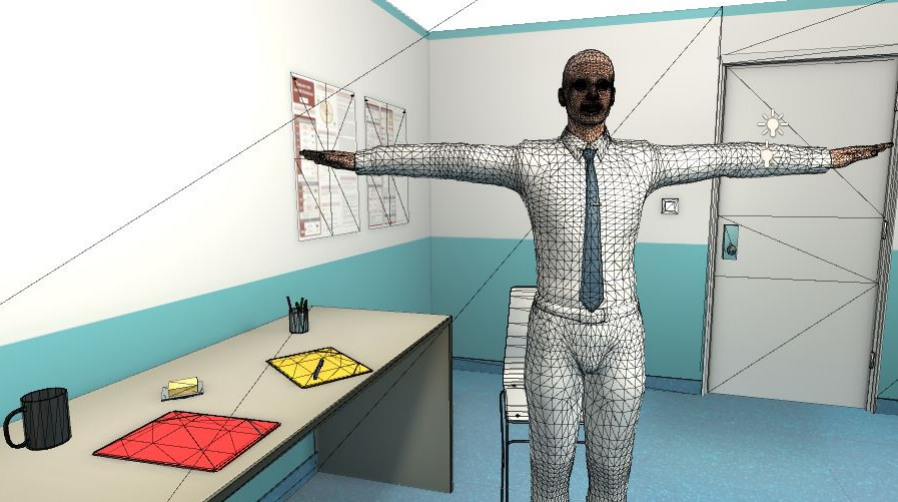
3D Virtual patient.

All the regular 3D content was created using 3Ds Studio Max (Autodesk), the leading 3D digital content creation solution (Figure 5). Figure 6 shows examples of the wireframes used in the creation of the 3D content. Wireframes are illustrations of the proposed VP simulation components and “assist in visual communication and design of the structure, functionality, learner interface, and positioning of an application” [24].

#### MedicActiV Platform

The MedicActiV platform [42] was designed to support digital training for health care providers and was used to host the simulated nursing scenario. The simulated scenario is a self-directed learning approach that is accessible via a computer or tablet. It is a 2D visual interface (Figure 4) with 3D content and is considered nonimmersive virtual reality [46]. The 2D elements include a graphical user interface, such as colors, icons, and text-based interfaces. The 3D elements are the dynamic *objects* created to have volume, such as the VP and the nurse’s office (Figure 6). MedicActiV is a software as a service solution that provides a library of virtual clinical cases. Thanks to the authoring tool, developers (eg, researchers, educators, health care providers) can easily create their own clinical case to share it with other users on the platform. The trainer can set up the training sessions’ parameters (eg, date, time, duration) and, immediately after any session, is able to retrieve several key performance indicators (eg, time, errors). Most of the clinical cases are available on multiple devices and operating systems (eg, Windows, macOS, iOS, Android). However, our clinical case with the HIV-positive VP was not available to everyone because it was embedded in a research process, and thus, was available only to a small sample of research participants, namely, the nurses.

#### 2.4. Ensuring Fidelity to Ensure Learner Engagement and Immersion Throughout the VP Simulation

One important feature to include in any simulation, be it virtual or not, is the concept of fidelity, also known as realism or authenticity [47]. Fidelity is about the learner’s perceptions of how well a simulation represents or reproduces reality [44] and offers them a realistic learning opportunity [15]. In this context, 3 modes of fidelity represent the ways humans think about reality: (1) physical; (2) conceptual (or semantic); and (3) emotional and experiential (or phenomenal) [44,45,48]. These modes can influence the learner’s engagement with and immersion into the simulation as well as their learning process [15,44,48].

The *physical mode* can be described by the *physical* properties of the VP (eg, movements, appearance) and of the nurse’s office, including objects and artifacts in that environment. The voice-over acting for the nurse and patient (eg, voice tone, vocabulary) are other elements of fidelity that were taken into account. The *conceptual* or *semantic mode* relates to concepts and their relationships, for example, theories, meaning, or information that are presented via various means (eg, text, voice-overs). It also involves the *if/then* relationship. In our simulated situation, it is globally represented by the following logic: if the nurse uses behavior change counseling techniques (communication skills) consistent with MI, this will open up a dialogue with the VP. If the nurse uses relational skills inconsistent with MI, then the VP will react accordingly (eg, defensively). Finally, the *emotional* and *experiential mode* refers to the learner’s emotions, feelings, and beliefs relating to their holistic experience of participating in the simulation. In Table 1, we present strategies used to ensure that the VP simulation had the characteristic of fidelity.

**Table 1:** Strategies used to ensure 3 modes of fidelity throughout the virtual patient simulation.

Modes of fidelity		Strategies used
Physical fidelity		Giving cues to the VP^a^ simulation team for their design of the VP: Taking a picture of a real nurse’s office for the representation of the virtual nurse’s office Adding objects that represent reality (eg, chart of antiretroviral therapy on the wall)
		Getting charts from real patient records to use the same vocabulary in the VP’s electronic record
		Designing the VP with human features (eg, facial expression and body movements) based on past experiences of the VP simulation team
		Using real voice-overs for both patient and nurse: Preselecting some French- and English-speaking actors, listening to their audio tracks and choosing the ones that best fit the spirit of the simulation Providing descriptive cues in the script besides the nurse’s and patient’s speech, so that the actors can respect the tone of voice and ambiance and vibe in the nurse-patient *virtual* relationship, consistent with SBN ^b^ and MI ^c^
		Getting written approval for each step of the graphical design before undertaking the subsequent one
Conceptual fidelity		Co-designing the clinical content with a nurse having expertise in HIV care and MI to ensure that the simulation reflects the nursing practice and the validity of the theory injected in preprogrammed interactions
		Meeting a clinical nurse specialist who is an expert in HIV care to discuss real-life situations of people living with HIV having difficulty taking their medication to identify nursing actions, in partnership with physicians and pharmacists, in nonadherence situations
		Validating the content with health care professionals
		Working with pedagogical engineering to make sure that good educational practices are met
Emotional and experiential fidelity		Promoting a positive learning experience and relatedness by creating messages that value and respect nurses’ competencies and current practice. In doing so, clinical content inventors must themselves be consistent with the MI values in their way of translating the educational content to the simulation
		Creating opportunities for reflection on action (virtual practice) by incorporating quizzes and feedback that represent what nurses do in their current practice
		Establishing a fiction contract with nurses [44,48]. The following message in the prebriefing video is intended to create such a contract: “The relational aspect of the practice of caring cannot be simulated to perfection. Needless to say, human beings are not preprogrammed to respond to a nurse this way or that. I invite you, then, to immerse yourself in this virtual simulation as if it were real and to pay attention to the interactions between Mr. Wilson and the nurse”.

^a^VP: virtual patient.

^b^SBN: strengths-based nursing.

^c^MI: motivational interviewing.

## Discussion

### Challenges and Strategies

Thus far, we have described VP simulation development methods and the deliverables of each phase and subphase, including the theory-informed content. We now discuss the main challenges and strategies used to overcome them.

The most salient challenges and strategies are discussed in this section. The second phase (ie, designing the content, sequence, and format of the VP simulation) was the most challenging because of the difficulty in determining realistic clinical content in a virtual learning environment. We had to preserve the philosophy and values of SBN and the collaborative partnership that shaped the nurse-patient relationship. We were also concerned with translating relational skills informed by MI within the simulated nurse-patient dialogue while ensuring that the functionalities of the VP platform could support the content. *We also had to find a balance between a nursing-driven approach and a technology-driven approach while optimizing the learning experience.*

### Challenge 1: Starting the Project With Divergent Conceptions of Virtual Patient Simulation Approaches

At the beginning of the project, the VP simulation team suggested a generic template that could help structure and organize the clinical content into pre-established *categories* consistent with virtual simulators already developed by the company. However, the 2 clinical inventors (GR and JP) felt that such a template would not be helpful because it reflected a problem-solving [47,49] or procedural [6] simulation approach. This is appropriate for teaching, for instance, clinical reasoning and diagnosis. To perform these tasks, learners must, for example, collect data and make diagnoses and treatment decisions based on their anamneses. In such simulations, learners are given a set of information from which they must draw conclusions (eg, in patient-facing problems with adherence to antiretroviral therapy, the nurse’s identification of solutions drives the direction of the VP simulation script). However, this approach did not mesh with a collaborative partnership as a form of the nurse-patient relationship, and thus with the nature of relational skills that are consistent with a narrative approach to simulation [47,49]. The ways of seeing the approach to the VP simulation diverged between the simulation team (who perceived it as a procedural one) and the clinical team (who perceived it as a narrative one).

The narrative approach, also called *situational simulation*, [6] is generally found in a personal storyline that progresses over time around a logic of cause and effect and that involves a decision-making process that yields different *outcomes* (or effects). The script is anchored in a strengths-based approach, consistent with MI, in which the virtual nurse’s role is that of a facilitator, using relational skills to open up the dialogue with the patient (eg, where a patient is facing problems with adherence to antiretroviral therapy, the identification of solutions *by the patient* drives the direction of the VP simulation script). At the time we started the VP simulation project, it would have been helpful to rely on the paper of Peddle et al [43] because it describes the development of VP to support undergraduate nursing students in learning nontechnical skills such as communication. The authors give an overview of the narrative approach and the *choose-your-own-adventure* game structure used to design the VP simulation.

#### Strategies: Getting a Mutual Understanding of the VP Simulation Approach That is Aligned With the Philosophy and Values of a Collaborative Nurse-Patient Relationship

We first consulted some virtual clinical cases on the MedicActiV platform to familiarize ourselves with the possibilities and limitations of the virtual learning environment and to understand how it works. It helped to understand how the VP simulation team perceived the simulated clinical situations and then pinpointed the differences between our different VP simulation approaches (procedural vs narrative). We collaborated closely with the project manager of the VP simulation team from the outset of the project. It was essential to make sure that the narrative approach, the SBN, the relational skills informed by MI, the principles of adult learning theories, and the functionalities of the simulation platform all meshed together. Holding regular meetings with the working committee allowed us to gain a better understanding of each other’s roles, responsibilities, and evolving perspectives on the VP simulation.

### Challenge 2: Struggling With the Complexity of Designing Realistic Relational Skills Into Preprogrammed and Simulated Nurse-Patient Dialogue While Preserving an Immersive Learning Experience

This challenge was three-fold: (1) translating complex actions (uptake of relational skills) within fully automated and preprogrammed nurse-patient dialogue, (2) having insufficient guidance in integrating such skills in this form of virtual simulation, and (3) designing the proper immersive and realistic 2D learning environment.

Writing the scripts with high-quality motivational responses is not just about wording. It is about creating a natural flow of interactions that involves great sensitivity and attention to verbal and nonverbal aspects of communication while respecting values such as empathy, collaborative partnership, acceptance, affirmation, and so on. Such an endeavor is easier to put into action spontaneously in real-time interactions. This challenge is well summarized by Villaume et al [50]: “While the processes and skills of MI are theoretically understandable, using them in individual utterances requires a considerable adjustment of vocabulary, grammar, emotional tone, and rhetorical strategy. Trying to work through these adjustments in real time with a standardized patient is difficult.”

Adding to the complexity of the actions to be performed is the lack of guidance on how to create and translate preprogrammed interpersonal nurse-patient interactions. It is easy to become quickly overwhelmed by the multiplicity of alternative scenarios and the growing decision tree (arborescence) if we do not limit the number of points at which learners can make choices and the number of options they have.

At the same time, as we were writing the script, SimforHealth had to design immersive and realistic nurse-patient interactions in a virtual learning environment, which was challenging. The decision tree is a kind of backbone for the nursing content, but it needs to be enhanced with graphic design, such as representing the *real appearance* of the VP’s digital facial expression and behavior. This is key to achieving a better commitment from the learners.

### Strategies: Creating a Collaborative and Work-in-Progress Writing Template

It is certain that we cannot make the nature of relational skills less complex, be they consistent with MI or not. However, the VP simulation’s clinical inventors, as educators, were accountable for being skilled with using MI to be able to transfer theoretical knowledge in an understandable way within the VP simulation and allow for an optimal learning experience. The quality of the VP simulation depends partly on the inventors’ expertise. In the working committee, the *codevelopment* involved knowledge transfer and an opportunity for discussion between these 2 members, given that one (JP) has expertise in MI whereas the other (GR) was new to this theory.

We had to build up our own method and find our own guidance to develop the VP simulation. The writing journey required a high level of creativity and inductivism. The flow of the storyline evolved over time, with a concern for making connections with theory and practice, so that nurses would benefit from a constructive and positive learning experience. In doing so, teamwork was the most important success element. To begin with, the 2 members of the working committee (GR and JP) worked in close collaboration to write the content for the nurse-patient consultation. We created a *writing template* (Multimedia Appendix 4) that evolved over time and represented a guide for both the clinical and the VP team. We wrote the script as a *choose-your-own-adventure* book, that is, by starting to write the green pathway, and then adding the red pathways. In addition, the role of learners’ emotions and feelings was taken into account. * From our perspective, the conception of the clinical content (scenario) had to depict the nursing practice (ie, what nurses actually do) without falling into stereotypes or judgments. The clinical content inventors themselves had to be consistent with MI principles and values in their way of translating the educational content to the simulation. Learners had to recognize themselves in the whole scenario (including traps and roadblocks) to be engaged with and immersed in the learning experience (fidelity). This principle is comparable to what Yardley et al [51] call “promotion of a learner’s positive experience and relatedness.”*

### Strengths of VP Simulation

We believe our VP simulation has a long life span because the action of adopting relational skills consistent with MI is *stable*, and does not need regular updates, as it would if the simulation focused on pharmacological treatment, where scientific breakthroughs occur frequently. Behavioral change counseling techniques (eg, asking open-ended questions, summarizing) are timeless. Another strength is that communication is a transversal skill used by all nurses, from novice to expert, and by all other health care professionals. Relational skills are then applicable independently of the health care professionals’ roles, the context of practice or level of experience, and of the population they care for. Focusing on this clinical behavior is a good avenue to ensure the adaptability, sustainability, and efficiency of this VP simulation. We ensured the integrity and quality control [52] of the MI intervention by relying on original work by the authors on MI [17] and by involving nurses who are experts in this approach: one nurse was involved in the design of the French content and a certified trainer in MI revised the English version. Furthermore, the involvement of a patient-partner added value at the early stage of the project, adding insight into the credibility and fidelity of the storyline and the graphic design of the VP simulation. It is a theory-informed intervention, and thus, we are confident in the quality of the clinical content and its potential to induce a practice change.

### Conclusions

The knowledge gained from our experience of VP simulation codevelopment has the potential to optimize the development process of other VP simulations, particularly those using a narrative approach. VP simulation is the only means to support learning; it is not the technology per se that generates learning [53]. Many elements were put together to create favorable conditions for generating a positive learning experience. We strongly believe that the duration of the codevelopment process can be shorter if developers have a clear idea of the theories to use to structure the VP simulation (eg, SBN, MI, adult learning theories) and if they know which approach fits (eg, narrative, problem-based) with their perspective of the clinical behavior or action to address. This paper offers concrete examples of how to translate behavior change counseling techniques derived from MI into an asynchronous and preprogrammed nurse-patient dialogue. The inductive approach used in codeveloping the content of the VP simulation was a transformative learning experience for the working committee.

With a view to professional development, nurses will have the opportunity to try this VP simulation, informed by experiential, theoretical, and empirical knowledge, and thereby help evaluate it. The standardized nature of the intervention is a strength of this approach that could be helpful for evaluation purposes. If the nurses’ relational competencies are enhanced, then the quality of the therapeutic relationship between nurse and patient may benefit and, ultimately, this can have positive repercussions on the health of people living with HIV.

## Appendix

Multimedia Appendix 1 Definitions and examples consistent with motivational interviewing.

Multimedia Appendix 2 Definitions and examples inconsistent with motivational interviewing.

Multimedia Appendix 3 Key elements of the VP simulation.

Multimedia Appendix 4 Excerpt of the final writing template.

Multimedia Appendix 5 Table of contents glossary.

Multimedia Appendix 6 Prebriefing video (an excerpt).

Multimedia Appendix 7 Demonstration of the virtual patient simulation (video).

## Abbreviations

2D: two-dimensional
CIHR: Canadian Institutes of Health Research
CPD: continuing professional development
FRQS: Fonds de Recherche du Québec–Santé
MI: motivational interviewing
SBN: strengths-based nursing
VP: virtual patient
