# Supplementary material for: Codeveloping a Virtual Patient Simulation to Foster Nurses’ Relational Skills Consistent With Motivational Interviewing: A Situation of Antiretroviral Therapy Nonadherence
Source: J Med Internet Res. 2020 Jul 15;22(7):e18225. doi: 10.2196/18225 (PMC7391166; doi:10.2196/18225)
Supplement: Multimedia Appendix 1 [file jmir_v22i7e18225_app1.docx]

**Multimedia Appendix 1**.

Definitions and concrete examples of relational skills that are consistent with the proficient use of motivational interviewing

| **Behavioural change counselling techniques** | **Definitions** | **Concrete examples of the simulated nurse-patient dialogue** |
| --- | --- | --- |
| **Asking open-ended questions** | The purpose of this type of question is manifold: to invite patients to reflect and elaborate; to gain an understanding of the patient’s frame of reference; to strengthen the collaborative/working relationship; to explore the situation in order to then find a clearer direction toward a possible change; to contribute to evoking the patient’s own motivation, goals and values; and to help plan the change goal. | Nurse: “What else is going on in your life these days?" |
| **Affirming** | Nurses place the emphasis on one of the patient’s positive aspects (strength, resource). | Nurse: “Yes, you do have experience and you stuck to your treatment very well for a long time.” |
| **Reflective listening** | Reflective listening is an essential MI^a^ skill in which reflection is used. A reflection is a nurse’s affirmation or hypothesis based on their understanding or interpretation of what the patient said, which then allows the patient to validate or adjust the meaning ascribed by the nurse. | (See the examples below) |
| Simple reflection | A simple reflection consists of repeating a patient’s words, remaining very close to what they say without adding any content or additional meaning. It is essentially an invitation to pursue or develop an idea further. | Patient: “I don’t want to change medication anymore… I'll be retiring soon, and I really want to enjoy it to the fullest.”  Nurse: “You want to be healthy in order to enjoy your retirement and to face all of your other responsibilities.” |
| Complex reflection | A complex reflection entails using the patient’s words and adding meaning or a hypothesis about what the person seems to be feeling. It can add impetus to the exploration process centred on the patient’s situation. | Patient: “I'm really busy at work. The stock market is constantly moving, I have a lot of meetings and there's staff turnaround too. We're very focused on performance and productivity. It's intense…”  Nurse: “You are overloaded at work lately. That causes you a lot of stress.” |
| Double-sided reflection | This sort of reflection serves to render both sides of the patient’s ambivalence explicit. Nurses might pick up what patients say as follows: “On the one hand, you’re saying that… and on the other hand, you’re saying that…” | Nurse: “On one hand, you would like to share your HIV status with your mother. It would make life easier and it would allow you to share what you're going through with her, which is important to you. On the other hand, it's not the right time. According to you, your mother is not ready to learn that you are HIV positive. And this is a time in your life when you do not feel that you can manage her reaction; the situation would be worse if she knew.” |
| **Summarizing** | Summarizing serves to expose patients to various elements that might be linked to one another. By presenting these elements in this manner, patients are afforded an overview of their experience. It is also a way of showing them that you are paying attention to what they say. | (See examples related to each type of summary) |
| Linking summary | This serves to tie elements together and to integrate one or more elements touched upon previously (during the same meeting or at a previous one). | Nurse: “So, to recap the situation, taking care of your mother is very important to you. You are doing everything you can to protect her wellbeing: even going as far as hiding your own health status to protect her.” |
| Collecting summary | This serves to dig deeper into the patient’s situation. | Nurse: "I understand you are very tired and stressed. Your work is very demanding with the stock markets fluctuating, meetings added on, a new co-worker to supervise, and all that on top of your regular duties. What else is going on in your life these days?" |
| Transitional summary | This serves to group together everything that seems important and to prepare a shift to another subject. | Nurse: “To summarize, first there's work which takes a lot of your energy and even more so these days because of the stock markets and having to supervise a new co-worker. Then there's your family which also takes a lot of your time: you are adjusting to living with your mother, and things are tense between you and your brother and sister. Your private life is also affected. Would you add anything to that? (open-ended question) |
| **Providing information and advice using the “Elicit-Provide-Elicit” approach** | Elicit-Provide-Elicit is a technique recommended in MI for exchanging information while affirming the patient’s expertise and autonomy. This technique requires asking the patient for permission to elaborate, for example, a change plan with him, seeing what will work for him, listening to him and getting his feedback. For example, providing reading materials and leaflets is a good intervention in and of itself as long as the patient has given his permission to do so, and that it corresponds to his needs.  The first “elicit” refers to all the questions that are put to patients PRIOR to providing any information. “Provide” corresponds to a small amount of information conveyed to patients, if necessary and with their permission. Finally, the second “elicit” aims to allow patients to integrate the new information into their decision-making process. | Nurse: “Is it all right if we go over your latest viral load results?” (*Asking permission*)  Patient: “Yes, because I am worried about that.”  Nurse: “What might cause a viral load to increase?” *(Elicit)*  Patient: “When the HIV becomes resistant to treatment. The medication doesn't work anymore, so the treatment has to be changed.”  Nurse: “Would you like me to share with you some tips and ideas which have been tried by other people living with HIV? (*Asking for permission*)  Patient: “Yes, I'm listening.”  Nurse: “Then it'll be up to you to determine which of these work best for you.”(*Autonomy*)  Patient: “Alright.”  Nurse: “Based on your experience, you could continue to set your alarm. You could leave a few pills at work in a different bottle than the original one, and leave them in a locked drawer. And at home, you could have some of the pills in your briefcase.”(*Provide*) |
| **Eliciting change talk by using confidence and change scales** | Using a scale from 0 to 10 allows patients to express themselves on, notably: perceived confidence in one’s ability to make changes (confidence ruler), and one’s willingness to make changes (readiness ruler and change ruler). | Nurse: “On a scale from 0 to 10, how confident are you in your ability to take your medication: 0 being not at all confident and 10 being totally confident?”  Patient: “I would say…6 out of 10.”  Nurse: “What makes you say 6 and not 2 out of 10?” |
| **Evoking a hypothetical change** | Evoking a hypothetical change allows patients to project themselves into a future different from the present and to imagine its advantages. Here is an example of such a nursing intervention. | Nurse: “Right now, you’re keeping your condition hidden from your mother. But let’s imagine she knew: What difference would it make?” |
| **Using guiding style of counselling** | The guiding style is the one preferred for MI. It stands midway between the directing style and the following style. Nurses who guide patients possess good listening skills and mobilize their expertise when necessary. Nurses nevertheless have a strategic goal when guiding patients in exploring their motivation for a potential change and their ability to make it. The spirit of a conversation about change should be more akin to dancing than to wrestling, whereas the former affirms the patient’s freedom to make their own choices. The guiding style is preferred by the virtual nurse in the digital simulation. | This guiding style is used throughout the virtual patient simulation by the use of relational skills that are consistent with MI. |

^a^MI: motivational interviewing
