# Supplementary material for: Codeveloping a Virtual Patient Simulation to Foster Nurses’ Relational Skills Consistent With Motivational Interviewing: A Situation of Antiretroviral Therapy Nonadherence
Source: J Med Internet Res. 2020 Jul 15;22(7):e18225. doi: 10.2196/18225 (PMC7391166; doi:10.2196/18225)
Supplement: Multimedia Appendix 2 [file jmir_v22i7e18225_app2.docx]

**Multimedia Appendix 2.**

Definitions and concrete examples of relational skills that are inconsistent with motivational interviewing -traps or roadblocks-

Healthcare providers can fall into these traps despite their best intentions to help their patients. A limitation of these written concrete examples is their inability to portray the friendly and respectful tone of voice of the nurses who want to support their patients.

| **Traps** | **Definitions** | **Concrete examples used in the simulated nurse-patient dialogue** |
| --- | --- | --- |
| **Counselling style of directing** | Nurses provide information and give advice without it being solicited by the patient. Implicitly, the directing counselling style implies “I know what you should do, and here’s how to do it.” [1] It is understood that patients who receive the information should follow the nurse’s (good) advice and comply. | Mr. Wilson has decided to keep his HIV status hidden from his mother. How does he plan to continue to take his medication as prescribed? In that case, if the nurse offers and provides solutions based on her experience, without first asking the patient how he plan to address this obstacle, then, this is an example of directive or prescriptive counselling style. |
| **Expert trap** | The nurse is the expert directing the structure and the content of the conversation. Mr. Wilson is invited (implicitly) to follow her lead. The nurse prioritizes what she feels is most problematic and offers resources without the patient expressly asking for them. There is a risk that the patient may disengage from the relationship, if not discontinue it entirely. | Patient: “I might have missed a dose... But it really doesn't happen often. It's human to forget sometimes. Even though it rarely happens...”  Nurse: “Even though it happens rarely, you have to try not to forget.” |
| **Righting reflex trap** | This is an instinctive intervention driven by good intentions on the part of nurses in favour of change (eg, for ART intake). However, “argue for one side and the ambivalent person is likely to take up and defend the opposite” [1]. When people are ambivalent about two options, the fact is that they already have two voices confronting one another inside their head (change talk and sustain talk). When nurses take a stand in favour of change within this “internal committee” (“You should do this… It is important that you understand that…”), they invite patients to justify themselves (“yes, but… it’s because…”). In a way, these nurses cede the floor to sustain talk and patients hear themselves go through reasons for maintaining the status quo instead of reasons for changing. Even when it is driven by the best intentions in the world, the righting reflex can elicit shame, anger and discouragement in patients. | As its name suggests, this is a reflex commonly used in the nursing practice, albeit in good faith, by nurses who wish to correct a problem. The nurse therefore suggests resources even though the patient hasn't asked for any. This may cause, on the part of the patient, resistance and/or disengagement from the therapeutic relationship. Rather than offering solutions to the perceived problems, it is preferable to first encourage the patient to talk about his experience and his needs. Solutions can be explored at a later time. |
| **Assessment trap** | Nurses with the best intentions ask questions in order to find out a load of information that will allow them, in their opinion, to help their patients. This puts nurses in an active role and patients in a passive role.  Insisting with an active assessment affords patients little opportunity to explore their motivation and their reasons (for example) for taking their medication and to share their own arguments in favour of this behaviour. The role of patients thus boils down to answering the questions put to them. This being said, assessments obviously fall within the scope of nursing practice and they are necessary. In certain emergency situations (eg, assessing risk for health deterioration or suicide risk), assessment is essential and fundamental. | Here are the key signs (or cues) of the assessment trap:   - Questions centre primarily on factual elements (eg, medication intake, intake conditions, omissions, side effects, conditions that might prevent medication absorption, interactions with other substances). - Questions are closed (did you, have you) or prompt patients to justify themselves (why…). |
| **Closed questions trap** | As an extension of the assessment trap, the purpose of closed questions is to obtain specific information. This type of question generally generates short answers. | Nurse: “If I understand correctly, you can't rely on them (brother and sister) for support?” |
| **Premature focus trap** | Nurses fall into this trap when they make an issue a priority, which is not necessarily a priority for the patient. Nurses insist on talking about the “real” problem at the expense of the patient’s priorities. Nurses do this with no ill intent, they just want to help. However, patients may disengage from the relationship and even put an end to the follow-up. | (*Mr. Wilson arrived at the nurse’s office a few minutes ago.*)  Patient: “I've had it up to here lately. It's really not the right time for that.”  Nurse: “Mr. Wilson, since I've known you a long time, I know you've already been through a period when your viral load was detectable because your medication intake was irregular. Would you say this is a similar situation?” |
| **Blame trap** | Nurses fall into this trap when they focus their intervention on the cause of the problem (e.g. detectable viral load, adherence questioned). They prompt patients to explain why the problem is so instead of asking them what could be done for things to be different. Patients tend to be defensive and may apprehend this dynamic even before the consultation | Nurse: “Your brother and sister are not very motivated to take care of your mother.” |
| **Chat trap** | Nurses and patients are just chatting beyond what is appropriate when the discussion takes place, leading to insufficient direction in the conversation. A large amount of “small talk” has little chance of having a positive impact on the individual changes a patient should make. This type of off-topic information should be kept within reasonable limits in the conversation. | Patient: “Well, I just have less time to think of everything, with work and taking care of my mother. I have a hectic schedule. I try to prioritize as best as I can but, let's just say I'm under a lot of pressure.”  Nurse: “Your situation reminds me of something else. If you don't mind, I'd like to make a slight digression before coming back to our conversation. My cousin works in finance. I agree that your working conditions are not reasonable.” |
| **Fear trap** | A message of fear can evoke sustain talk from the patient [1], which in turn can lead them to the path of avoiding rather than approaching change (inspired by [2]. | Patient: “My viral load is 1000. I just can't believe it…You remember: before my treatment changed, my viral load had been undetectable for 6 years…”  Nurse: “It is important to act fast to reduce the viral load. As you know there is a risk of resistance linked to a detectable viral load. In this case, changing the treatment is sometimes necessary.” |

**References**

1. Miller WR, Rollnick S. Motivational interviewing: Helping people to change. 3rd ed. New York, NY: The Guilford Press; 2013. ISBN:978-1-60918-227-4

2. Godin G. Les comportements dans le domaine de la santé : comprendre pour mieux intervenir. Montréal: Presses de l’Université de Montréal; 2012. ISBN:978-2-7606-2779-6
