# Supplementary material for: Codeveloping a Virtual Patient Simulation to Foster Nurses’ Relational Skills Consistent With Motivational Interviewing: A Situation of Antiretroviral Therapy Nonadherence
Source: J Med Internet Res. 2020 Jul 15;22(7):e18225. doi: 10.2196/18225 (PMC7391166; doi:10.2196/18225)
Supplement: Multimedia Appendix 3 [file jmir_v22i7e18225_app3.docx]

**Multimedia Appendix 3.**

Key elements of the virtual patient simulation

| **Intervention** |  |  |
| --- | --- | --- |
| **Elements** | **Subelements** | **Descriptor** |
| Simulation codevelopment | Duration | The codevelopment of the VP^a^ simulation was undertaken over a 16-month period [August 2017 to December 2018]; the English adaptation, over a six-month period [December 2018 to June 2019]. |
| Simulation approach | Simulation type | Narrative approach [1] or situational e-simulation [2]. |
|  | Simulation modality – Virtual patient | A Web- and screen-based program, available on computer or tablet, that allows learners to interact with a two-dimensional animated human character called the “virtual patient.” Learners emulate the role of the healthcare providers, within a first-person perspective. |
|  | Simulation functionality | Some choices made by the learners in the simulation impact the virtual patient’s reaction/response (cause and effect), and then the flow of the nurse-patient conversation, while other choices don’t bring any change. Learners can make many mistakes and are always invited to make different choices to go back to the “good” pathway. |
|  | Simulation access | Learners have to create an account on the MedicActiv platform. In doing so, a secured URL was given to the nurses to allow them to create an individual account. The simulation is not accessible via mobile phone. |
| Learner orientation | Orientation to the simulation environment | As a self-directed learning process that is provided individually, the introduction (prebriefing) to the simulation is video and text based. The video introduction is facilitated by the nursing student–researcher in charge of the project. Written information is given to orient the learners’ navigation on the MedicActiv platform. |
| Simulation environment | Location | It is planned that nurses can use the simulation in a convenient location (eg, from the workplace, home). |
|  | Equipment | The virtual patient simulation is available in two versions: 1) online, and 2) an application.  Both versions are accessible with computer/laptop or tablet devices with stable internet connectivity with additional audio-visual display equipment. The application version supports higher-quality graphics than the online version does. |
| Stimulation event script | Event description | The simulation is supported by written instructions to guide the learner’s navigation. |
|  | Learning objectives | - Spot traps in nursing interventions that can shut down communication with the patient and that enable sustain talk. - Identify and apply nursing interventions that optimize openness to the patient’s experience. - Identify cues in the patient’s speech that reflect change talk. - Choose and apply nursing interventions that elicit change talk. - Describe the principles consistent with motivational interviewing that structure information sharing with the patient. - Target the key elements that are important to include in providing information to the patient. - Identify principles to build an action plan with the patient. |
|  | Mode of evaluation | Within the MedicActiv platform, a functionality allowing a choice between two evaluation modes: 1) summative evaluation, with a final “score,” in which learners cannot change their choices/answers during the learning activity; 2) formative evaluation, which allows learners to change their answers throughout the simulation. These evaluation modes may impact the learners’ experience during the simulation. We didn’t want learners to feel as if they were in an “exam period,” but rather we wanted to create a constructive environment and a low level of stress. We set functionalities so that nurses can change their answers to the quizzes throughout the simulation to allow them to learn from their mistakes. This formative evaluation is in line with the clinical team’s vision of continuing professional development. |
|  | Preparation of actors | Voice-overs of the virtual nurse and patient were produced by French- and English-speaking professional actors. They were guided to use a respectful, empathic, nonjudgmental tone of voice and to share feelings in an authentic way (eg, patient’s concerns) for both communication skills, consistent or not with motivational interviewing. All dialogues were scripted. |
| Exposure | Duration | There is one consultation session. The duration of the VP simulation is expected to vary depending on: the good/bad choices made by the learners; the time spent carefully reading the feedback and additional information materials (such as the glossary). The duration is around 45 minutes. |
| Participant group | Frequency/repetitions | Learners may stop their participation at any time and then reconnect to continue at the same place as they left off the previous time. |
|  | Predictability of simulation | The VP simulation is consistent with predictable storyline progression based on branching algorithms across all learners. The story has the same end for everyone. |
|  | Learner characteristics | The simulation is applicable to all nurses (and potentially healthcare providers), from novice to expert, as long as they have minimal computer literacy skills. |
|  | Learner roles | The learner is compelled to act as 1) an observer of the situation, in which the virtual nurse may be seen as a role model, and 2) an active participant. |
| Simulation element design | Introduction: prebriefing | The prebriefing video (<https://youtu.be/hE4oVY-EZ7c>) lasts 12 minutes and contains seven sections: 1) nursing student–researcher’s background and a summary presentation of the project; 2) qualitative research findings based on HIV nurses’ experiences (origin of the project); 3) definition and functioning of the VP simulation; 4) introduction to motivational interviewing; 5) four cores values (or the spirit) of motivational interviewing; 6) general learning objectives; and 7) invitation to be on the lookout for certain interactions between the virtual nurse and the patient. |
|  | Patient’s electronic record | Learners are invited to look at the six rubrics of the patient’s electronic record before starting the consultation: 1) psychosocial history and vulnerability factors; 2) lifestyle; 3) HIV history; 4) medication history; 5) clinical notes from the microbiologist and nurse; and 6) purpose of the consultation. |
|  | Full script of the nurse-patient consultation | The script is divided into the four processes (Table 2) of motivational interviewing: 1) engaging; 2) focusing; 3) evoking; and 4) planning. Multimedia Appendix 5 contains an excerpt of the full script. |
|  | Fidelity and its modes: physical/conceptual/  emotional and  experiential | See the section “Modes of fidelity to ensure learners’ engagement and immersion in the VP simulation.” |
|  | Quizzes | There are 14 quizzes, allowing learners to reflect on the most appropriate intervention to elicit change talk and to open up on the patient’s experience. Most of questions are multiple-choice, while a few are open-ended. The quizzes are practice-based: “what would you do in this case and how would you respond to Mr. Wilson?” Usually, there is only one good answer (green pathway) consistent with motivational interviewing and a couple of traps (red pathway) that are often used reflexively by healthcare providers. |
|  | Feedback – Timing | Synchronous written feedback provided right after the learner has made a choice (quiz). |
|  | Feedback – Source | Feedback mechanisms are organized in two ways: 1) the learners’ answer following a quiz does not change the flow of the nurse-patient conversation because learners have to redo the quiz until they select the right answer; 2) the learners’ answer changes the flow of the dialogue and then they have to observe the effect of the chosen intervention on the patient’s reactions and speech. After a short observation period, learners are invited to redo the quiz until the right answer is chosen. |
|  | Feedback – Content | The structure of the written feedback is: 1) the name of the communication skill or behaviour change counselling technique or trap (eg, directive counselling style); and 2) the reason why this intervention is consistent or not with motivational interviewing, and the consequences of using it in relational (dis)engagement with the patient. |
|  | Visual cues – green and red labels | Labels are represented with green or red insets that appear during the dialogues. A green label means the learner is on the right track, that is, the intervention is appropriate; a red label means it’s not. In other words, these labels, developed specifically for this project, are a form of constructive feedback. |
|  | Glossary | The glossary is a supplementary electronic educational material containing 20 pages on motivational interviewing theoretical concepts, definitions and applications, with concrete examples from the simulation (see Table of Contents in Multimedia Appendix 5). |

^a^ VP: virtual patient

**References**

1. Bearman M. Is Virtual the Same as Real? Medical Students’ Experiences of a Virtual Patient. Acad Med 2003;78(5):8. [doi: 10.1097/00001888-200305000-00021]

2. Cant RP, Cooper SJ. Simulation in the Internet age: The place of Web-based simulation in nursing education. An integrative review. Nurse Educ Today 2014 Dec 1;34(12):1435–1442. [doi: 10.1016/j.nedt.2014.08.001]
