# Supplementary material for: Codeveloping a Virtual Patient Simulation to Foster Nurses’ Relational Skills Consistent With Motivational Interviewing: A Situation of Antiretroviral Therapy Nonadherence
Source: J Med Internet Res. 2020 Jul 15;22(7):e18225. doi: 10.2196/18225 (PMC7391166; doi:10.2196/18225)
Supplement: Multimedia Appendix 4 [file jmir_v22i7e18225_app4.docx]

**Multimedia Appendix 4.**

Excerpt of the final writing template

| **Engaging PROCESS** | **Feedback (including green & red labels)** |
| --- | --- |
| (IN BLUE: AUTOMATED DIALOGUE)  Introduction | |
| Nurse: Hello Mr. Wilson. How are you? |  |
| Patient: (*Visibly bothered or worried*): I'm OK…but I'm in a bit of hurry. |  |
| Nurse: The doctor spoke to you about the results of your viral load and CD4 count. |  |
| Patient *(Visibly flabbergasted*): Yeah…My viral load is 1000. I just can't believe it…You remember: before my treatment changed, my viral load had been undetectable for 6 years… (*silence*) |  |
| **# 1. QUIZ (single choice question)–React to detectable viral load**  **Which of these interventions seems most appropriate to you in response to Mr. Wilson's remarks? *"My viral load is 1000. I just can't believe it…You remember: before my treatment changed, my viral load had been undetectable for 6 years…"***  For additional information refer to the glossary.  Note for VP simulation team: When user clicks on one option or the others, the automated dialogue starts, attached with the corresponding label, and the written feedback is given. | |
| Answer 1: (*Reassuring, soothing tone^a^*)  You really weren't expecting this. You are shocked by the results of your viral load this morning. (True) | ✓ **Complex reflection** (Good answer)  The style of communication used aims to guide the conversation. A complex reflection is a way of adding meaning or hypothesis to what Mr. Wilson said.  Click on "Continue" to return to dialogue. |
| Answer 2: (*Reassuring, soothing tone*)  I can imagine it's difficult to follow a new course of treatment. But don't worry, many people living with HIV go through this. I can suggest some tips. (False) | **X Directive communication style and expert trap**  The directive communication style places the nurse in the role of the expert. The patient is (implicitly) invited to follow her lead. There is little room given for the patient's own experience.  Select another answer ("Continue") which aims to explore Mr. Wilson’s reaction to the results of his viral load. |
| Answer 3: (*Nurse’s reassuring tone: Wants to express to the patient that there is no need to feel threatened)*  I know you were undetectable for a long time. And actually, we are going to look at ways to get you back to being undetectable. You're in the right place and I'm going to help you. Let's start by discussing how you take your medication. (False) | **X Directive communication style and expert trap**  The directive communication style places the nurse in the role of the expert. The patient is (implicitly) invited to follow her lead. There is little room given for the patient's own experience.  Select another answer ("Continue") which aims to explore Mr. Wilson’s reaction to the results of his viral load. |
| Answer 4: (*Reassuring tone*)  It is important to act fast to reduce the viral load. As you know there is a risk of resistance linked to a detectable viral load. In that case, changing the treatment is sometimes necessary. (False) | **X Fear trap**  This message can cause a sense of fear leading the patient on the path of avoidance rather than change.  Select another answer (“Continue”) which aims to explore Mr. Wilson’s reaction to the results of his viral load. |
| Note for the VP simulation team: Go back to automated dialogue following quiz no. 1 (Mr. Wilson overwhelmed by the situation) | |
| Patient: *(overwhelmed by the situation)* No…well…It's just that…I've had it up to here lately. This really isn't the right time for that. |  |
| The "Engaging" process is completed. Now choose the next step, "Focusing", to continue the interview. | |
| **FOCUSING PROCESS** | **FEEDBACK (INCLUDING GREEN & RED LABELS)** |
| **#2. QUIZ – Opening up about patient's experience (single choice question)**  **In the case of Mr. Wilson’s clinical picture, it seems appropriate to address the topic of medication with him. The following options are all designed to address this topic one way or another. However, taking into account what Mr. Wilson has said: *"I've had it up to here lately. It's really not the right time for that",* which would be the priority action to take to most likely encourage him to open up about what he's going through?** | |
| Answer 1: First, I have to reassure Mr. Wilson and normalize the fact that he has a lot to handle, by saying for example: "I do understand, we are all very busy this time of year! But don't worry, the holidays will soon be here." (False) | **X** **Traps – False reassurance, non-recognition of emotions**  In response to Mr. Wilson' emotional remarks, rational arguments are stated. This is a form of normalization of emotions.  Select another answer ("Continue") which aims to encourage Mr. Wilson to open up about his experience |
| Answer 2: I should first assess the possible causes that would explain his blood test results. For example: "Mr. Wilson, allow me to go over with you what could be causing your viral load to be detectable." (False) | **X Trap – Assessment**  Beginning a consultation by assessing the possible causes of a detectable viral load places the nurse in the role of the expert, and the patient in a passive role which can jeopardize the engagement in the relationship.  Select another answer ("Continue") which aims to encourage Mr. Wilson to open up about his experience |
| Answer 3: First, I need to understand and explore the patient's concerns. I can pick-up from Mr. Wilson' comment: "It's really not the right time for that." (True) | **✓ Simple reflection**  The use of simple reflection consists in repeating the patient's last words or statement to allow them to go on with the conversation. Mirroring the preceding emotion expressed by Mr. Wilson, rather than immediately attempting to find causes to the increased viral load, improves the chances of opening up the dialogue.    Continue the dialogue ("Continue") and pay close attention to Mr. Wilson’s remarks. |
| Answer 4: First, I must subtly approach the topic of treatment adherence as the main cause of his detectable viral load: "Mr. Wilson, since I've known you a long time, I know you've already been through a period when your viral load was detectable because your medication intake was irregular. Would you say this is a similar situation?" | **X Premature focus trap, blaming trap, directive style trap, expert trap**  Premature focus arises when focusing on and determining too quickly the purpose of the consultation. Despite her best intentions, the nurse prioritizes what she sees is most problematic for Mr. Wilson (addressing his viral load), when in fact he has not made it a priority. There is a risk the patient may disengage from the relationship, if not discontinue it entirely.  Select another answer ("Continue") which aims to encourage Mr. Wilson to open up about his experience |

^a^ This is an example of guidance provided to actors for the recording of voice-overs. Learners don’t see this type of comment.
