# Supplementary material for: Codeveloping a Virtual Patient Simulation to Foster Nurses’ Relational Skills Consistent With Motivational Interviewing: A Situation of Antiretroviral Therapy Nonadherence
Source: J Med Internet Res. 2020 Jul 15;22(7):e18225. doi: 10.2196/18225 (PMC7391166; doi:10.2196/18225)
Supplement: Multimedia Appendix 5 [file jmir_v22i7e18225_app5.docx]

**Multimedia Appendix 5.**

Table of contents of the glossary

Motivational interviewing

What it is (definition)

What it is not

Ambivalence

Change talk

Sustain talk

Importance, confidence, readiness, and change rulers

Evoking a hypothetical change

Counselling styles: directing, guiding, following

Four processes in motivational interviewing

Engaging

Focusing

Evoking

Planning

Five relational skills essential for the proficient practice of motivational interviewing

1) Asking open-ended questions

2) Reflective listening

Simple reflection

Complex reflection

Double-sided reflection

3) Summarizing

Linking summary

Collecting summary

Transitional summary

4) Affirming

5) Providing information and advice using the “Elicit-Provide-Elicit” approach

Traps that can promote relational disengagement

Confidence and conviction mismatch trap

Blame trap

Assessment trap

Chat trap

Premature focus trap

Closed question trap

Fear trap

Righting reflex trap

Table 1. Assumptions underlying information exchange that are consistent and inconsistent with motivational interviewing

Table 2. The “Elicit-Provide-Elicit” approach

Table 3: Distinction between confidence and conviction
